# Bevacizumab plus dacomitinib combination therapy for L858R‐mutated metastatic lung adenocarcinoma: A report of two cases

**DOI:** 10.1111/1759-7714.14396

**Published:** 2022-03-27

**Authors:** Chi‐Kang Teng, Chieh‐Lung Chen, Ting‐Han Chen, Wen‐Chien Cheng, Chih‐Yen Tu

**Affiliations:** ^1^ Division of Pulmonary and Critical Care Medicine, Department of Internal Medicine China Medical University Hospital Taichung Taiwan; ^2^ School of Medicine China Medical University Taichung Taiwan

**Keywords:** angiogenesis inhibitors, bevacizumab, combined modality therapy, dacomitinib, epidermal growth factor receptor

## Abstract

Dual inhibition of the epidermal growth factor receptor (EGFR) and vascular endothelial growth factor pathways for the treatment for *EGFR*‐mutated, metastatic non‐small cell lung cancer is supported by previous randomized controlled trials. However, the use of second‐generation irreversible EGFR tyrosine kinase inhibitor (TKI) dacomitinib in combination with antiangiogenic therapy has not been reported in the literature. Here, we report the case of a 73‐year‐old man who presented with hemoptysis and dyspnea on exertion and was diagnosed with right upper lung adenocarcinoma with pleural metastasis and L858R mutation. The second case is of a 60‐year‐old woman who presented with low back pain and was diagnosed with right lower lung adenocarcinoma with bone metastasis and L858R mutation. Both patients underwent first‐line therapy with the TKI dacomitinib in combination with bevacizumab. The first patient showed a nearly complete response, and the second patient showed a partial response after the combination therapy and no severe side effects.

## Graphical abstract

Bevacizumab plus dacomitinib combination therapy enabled two patients with untreated NSCLC harboring L858R mutation of exon 21 to achieve a good response without obvious side effects, which might be recommended as first‐line therapy.

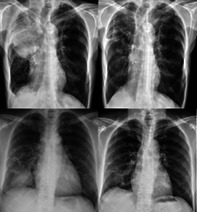

## INTRODUCTION

Dual inhibition of the epidermal growth factor receptor (EGFR) and vascular endothelial growth factor pathways in patients with *EGFR*‐mutated, metastatic non‐small cell lung cancer (NSCLC) has previously been reported in randomized controlled trials (RCTs), including RELAY,^1^ JO25567,^2^ and NEJ026,^3^ to increase progression‐free survival (PFS). These RCTs were limited to the use of erlotinib, a first‐generation EGFR tyrosine kinase inhibitor (TKI), in combination with ramucirumab^1^ or bevacizumab.^2^, ^3^ A real‐world study reported that the combination of afatinib, a second‐generation EGFR TKI, and bevacizumab is an effective therapy.^4^ However, to the best of our knowledge, no published report has so far discussed the efficacy and safety of dacomitinib, another second‐generation irreversible EGFR TKI, in combination with antiangiogenesis therapy. Here, we report two patients with L858R‐mutated, metastatic lung adenocarcinoma who gained benefits from receiving dacomitinib and bevacizumab combination therapy and experienced no severe side effects.

## CASE REPORT

### Case 1

**FIGURE 1:**
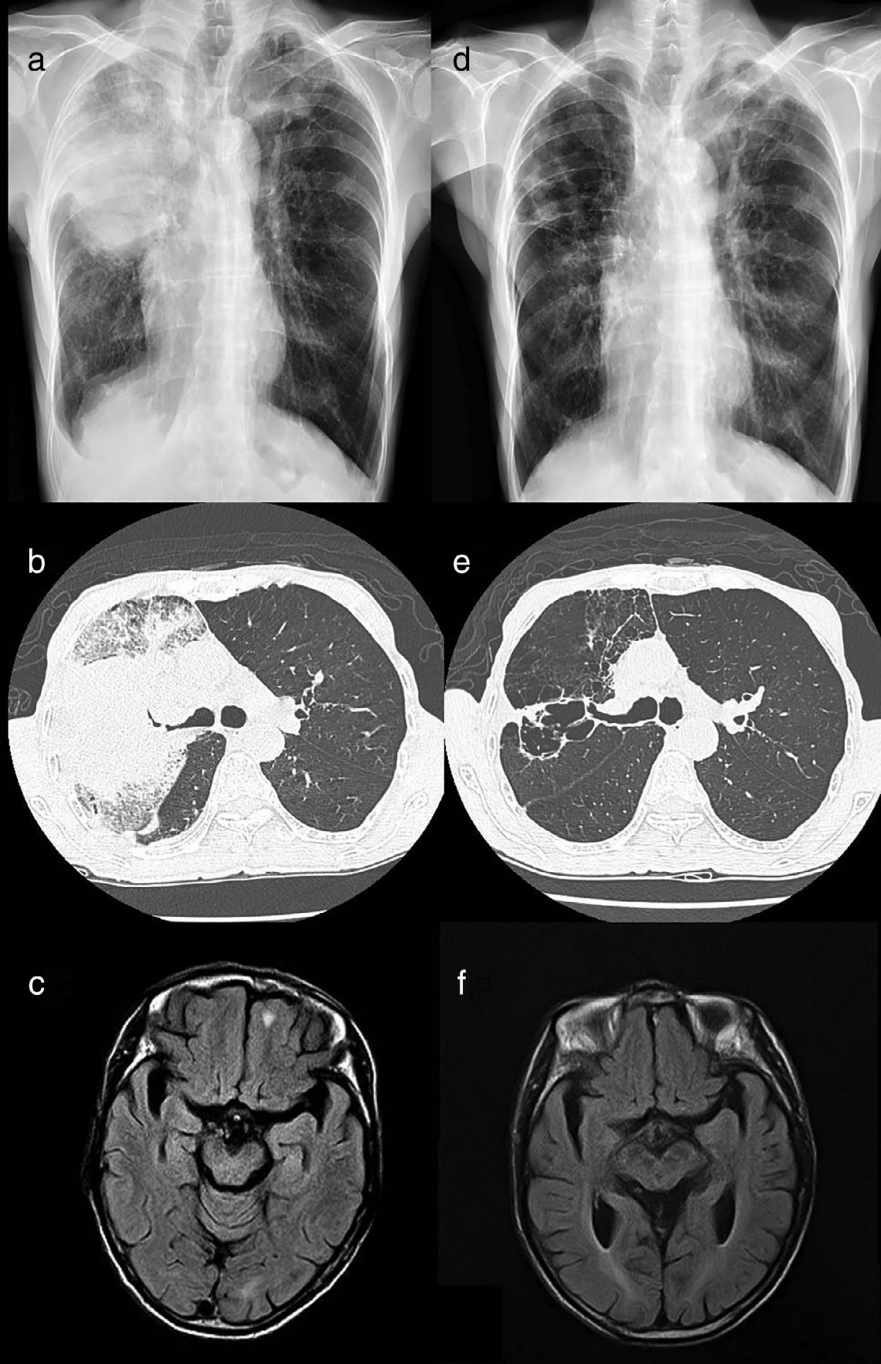
Findings of chest radiograph, computed tomography (CT), and brain magnetic resonance imaging (MRI) performed before and after the administration of combination therapy. (a) A huge mass in the right upper lung (RUL) with right pleural effusion was observed on the chest radiograph. (b) A huge mass in the RUL of up to 10.6 cm in diameter with necrotic components and narrowing of the RUL bronchus was visible on chest CT. (c) One metastatic tumor was observed in the left inferior frontal lobe before the administration of combination therapy. (d) The size of the RUL mass significantly decreased, and no pleural effusion was found on chest radiograph. (e) The primary RUL mass had significantly decreased in size and exhibited multiple cavities on chest CT. (f) Complete remission of the metastatic tumor in the left inferior frontal lobe was observed after administration of combination therapy

A 73‐year‐old man visited our hospital complaining of hemoptysis for 1 week accompanied by dyspnea on exertion. Chest radiography and computed tomography (CT) revealed a large mass in the right upper lung (RUL) with a diameter of approximately 10.6 cm (Figures 1a,b), mild right pleural effusion with pleural calcification, and enlarged lymph nodes in the right paratracheal and hilar regions. Additionally, brain magnetic resonance imaging (MRI) revealed a metastatic lesion in the left frontal lobe (Figure 1c). The patient subsequently underwent bronchoscopic endobronchial ultrasound biopsy and was diagnosed with stage IV lung adenocarcinoma with L858R point mutation in exon 21.

He received first‐line therapy with dacomitinib (30 mg, daily) plus bevacizumab (7.5 mg/kg, every 3 weeks). The results of follow‐up imaging performed after 3 months showed decreased primary tumor size in chest CT (Figure 1d,e) and improved brain metastasis in brain MRI (Figure 1f). There were also no obvious side effects, except mild skin rash development.

### Case 2

**FIGURE 2:**
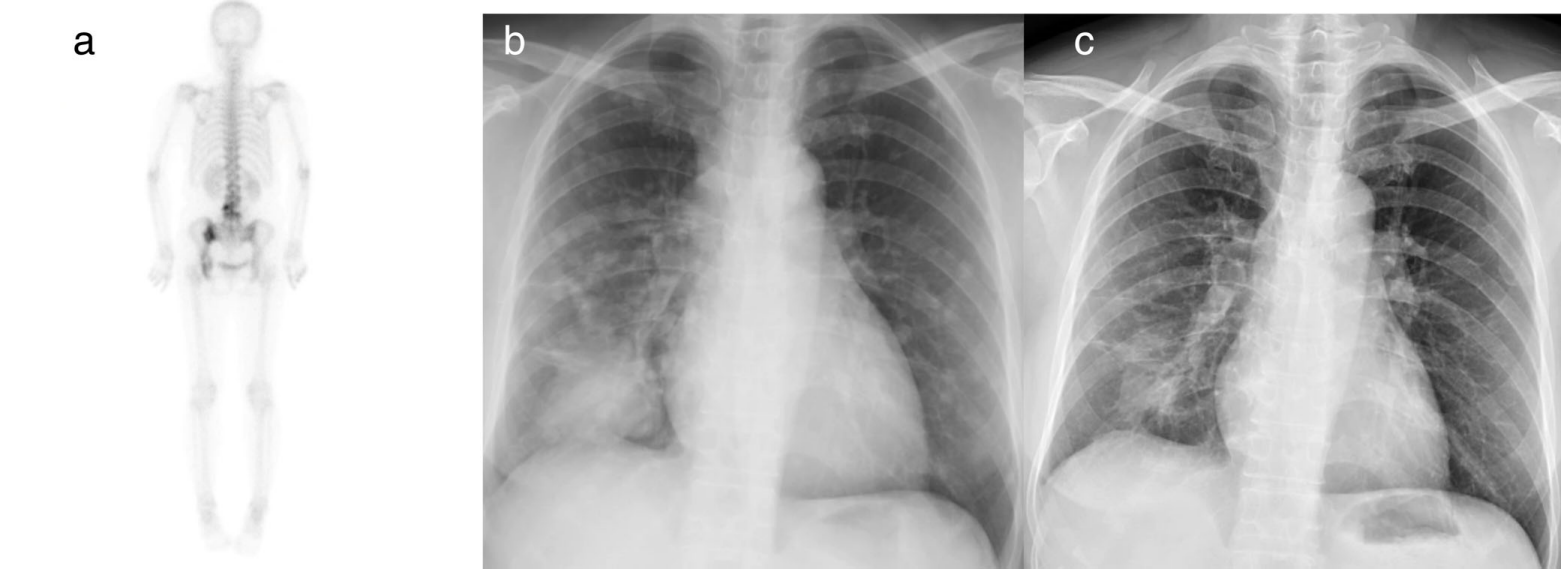
Findings of bone scan and chest radiography performed before and after administration of combination therapy. (a) Bone scan findings showed metastases at the lumbar spine. (b) A huge mass in the right lower lung (RLL) with lung‐to‐lung metastasis was observed on chest radiograph. (c) Chest radiograph revealed the RLL mass and bilateral lung nodules had significantly decreased in size

A 60‐year‐old woman visited our hospital with a complaint of low back pain for 2 months accompanied by chronic cough. Chest radiography and bone scan demonstrated a mass in the right lower lung (RLL) as well as lung‐to‐lung and lumbar spine metastases (Figures 2a,b). She was also diagnosed with stage IVb lung adenocarcinoma with spine metastasis and L858R point mutation in exon 21. Dacomitinib (30 mg daily) plus bevacizumab (7.5 mg/kg, every 3 weeks) was administered as the first‐line therapy. Follow‐up chest radiography performed after 2 months showed a significantly decreased primary tumor size. There were also no obvious adverse effects, except self‐limited mild epistaxis and skin peeling on the feet.

After 6 months of undergoing therapy, both patients remained stable with no signs of disease progression.

## DISCUSSION

Here, we report two cases of metastatic lung adenocarcinoma with driver gene L858R mutation. The patients received first‐line therapy with dacomitinib plus bevacizumab and showed an excellent response to it. No obvious severe adverse effects were noted.

Lung cancer, especially NSCLC, is the leading cause of cancer‐related death worldwide. The development of EGFR TKIs has markedly improved the treatment of advanced NSCLC. Approximately 80%–90% of patients with *EGFR*‐mutated NSCLC will have either an exon 19 deletion or a common L858R point mutation. Both these mutations are sensitive to EGFR TKIs. However, several studies have previously reported that patients with exon 19 deletions treated with EGFR TKIs had better outcomes compared with those with L858R substitutions.^5^, ^6^ Therefore, NSCLC caused by the two mutations should be treated as distinct types of NSCLC.

Several studies have proved that the combination therapy comprising EGFR TKI and antiangiogenesis therapies has an important role in improving PFS. Both RELAY and NEJ026 disclosed that erlotinib plus antiangiogenesis therapy resulted in longer PFS compared with erlotinib alone as the first‐line therapy for EGFR‐mutated, metastatic NSCLC.^1^, ^3^ The combination of EGFR TKI and antiangiogenic drugs seems to be more beneficial for patients with L858R mutation compared to those with exon 19 deletion.^1^, ^3^

Dacomitinib is an irreversible pan‐HER TKI that targets the kinase domains of EGFR, ErbB2, and ErbB4 of the EGFR signaling pathway. As an irreversible inhibitor, dacomitinib exerts pharmacodynamic effects for a longer duration compared with first‐generation TKIs, which were considered as the alternative first‐line therapy for *EGFR*‐mutated NSCLC in the ARCHER 1050 trial.^7^ Furthermore, subgroup analysis with exon 21 L858R mutation showed that the median overall survival for patients receiving dacomitinib was 32.5 months, whereas that for those receiving gefitinib was 23.2 months.^8^ Nonetheless, compared with gefitinib, dacomitinib has a higher rate of side effects.^7^ According to these studies, dacomitinib and antiangiogenesis therapy could provide a survival benefit to patients with L858R mutation, which is worthy of further discussion.

In this study, the patients showed a good response to combination therapy with dacomitinib and bevacizumab with a reduced standard dose without obvious severe side effects, which was comparable with previous studies on the combination of EGFR TKI and antiangiogenesis therapies. The standard dose of dacomitinib is 45 mg per day, whereas bevacizumab should be administered at a dose of 15 mg/kg for 3 weeks, according to previous RCTs.^1^, ^2^, ^3^, ^7^ However, both patients in this study received 30‐mg/day dacomitinib plus 7.5 mg/kg bevacizumab every 3 weeks, as the first‐line therapy. A previous study showed that the 7.5‐mg/kg dose was as effective as the 15‐mg/kg dose when used in combination with chemotherapy, for the treatment of patients with nonsquamous NSCLC.^9^ In a recent real‐world study conducted in Taiwan, 57 patients received afatinib and bevacizumab as the initial therapy; of these, 56 patients (98.2%) received a dose of 7.5‐mg/kg bevacizumab for each cycle mainly because it is not being covered by the National Health Insurance in Taiwan. A median PFS and an overall survival of 23.9 and 45.9 months, respectively, were observed, suggesting that a reduced bevacizumab dose offered an efficacy similar to that provided by the full dose.^4^ Another real‐word study demonstrated that an initial dose of 30‐mg dacomitinib also provided disease control benefits.^10^ In the current report, the patient in case 1 had a bodyweight of only 33 kg, and both patients received dacomitinib and bevacizumab combination therapy. Therefore, the patients received 30 mg of dacomitinib per day plus the 7.5 mg/kg dose of bevacizumab every 3 weeks as the initial therapy, to prevent the occurrence of severe side effects. During the treatment course, the two patients did not have diarrhea, stomatitis, or fatigue; however, they experienced mild acneiform skin rash, skin peeling, and mild self‐limited epitaxis.

Moreover, the novel combination therapy demonstrated clinically meaningful efficacy against brain metastases in the first case. Although the phase 3 ARCHER‐1050 study did not assess the efficacy of dacomitinib in patients with brain metastasis, several studies have reported that dacomitinib demonstrates efficacy in improving the central nervous system of patients with EGFR TKI‐naïve *EGFR*‐mutated NSCLC in actual clinical settings.^10^, ^11^ The outcome of the first case described in this study support the aforementioned findings. Both patients are currently stable after 6 months of therapy. Although the follow‐up time was around half a year, we wanted to report this novel combination therapy as soon as possible, for which no existing reports are available, to the best of our knowledge.

In conclusion, our patients with L858R‐mutated, metastatic lung adenocarcinoma showed an excellent response to this novel combination therapy, with no obvious side effects. However, sufficient follow‐up is essential to confirm whether this combination therapy resulted in a longer PFS compared with dacomitinib monotherapy. Further RCTs are needed to establish whether dacomitinib plus antiangiogenic drugs can be a standard therapy for *EGFR*‐mutated metastatic NSCLC.

## CONFLICT OF INTEREST

The authors declare no conflict of interest.
